# Case report: Sideroblastic anemia with B-cell immunodeficiency, periodic fevers, and developmental delay: Three cases and a literature review

**DOI:** 10.3389/fped.2023.1001222

**Published:** 2023-03-02

**Authors:** Xiangyuan Chen, Fang Fu, Xiaolan Mo, Suyun Cheng, Huasong Zeng

**Affiliations:** ^1^ Department of Allergy, Immunology and Rheumatology, Guangzhou Women and Children's Medical Center, Guangdong Provincial Clinical Research Center for Child Health, Guangzhou Medical University, Guangzhou, China; ^2^ Department Institute of Birth Health and Perinatal Medicine, Guangzhou Women and Children's Medical Center, Guangdong Provincial Clinical Research Center for Child Health, Guangzhou Medical University, Guangzhou, China; ^3^ Department of Pharmacy, Guangzhou Women and Children's Medical Center, Guangdong Provincial Clinical Research Center for Child Health, Guangzhou Medical University, Guangzhou, China

**Keywords:** SIFD, TRNT1, immunodeficiency, periodic fever, developmental delay

## Abstract

Sideroblastic anemia with B-cell immunodeficiency, periodic fevers, and developmental delay (SIFD) is a serious autosomal recessive syndrome caused by biallelic mutations in cytosine–cytosine–adenosine tRNA nucleotidyltransferase 1 (*TRNT1*). The main clinical features of SIFD are periodic fevers, developmental delay, sideroblastic or microcytic anemia, and immunodeficiency. Herein, we report three cases of SIFD with compound heterozygous variants of *TRNT1*. Patients 1 and 2 were siblings; they presented with periodic fevers, arthritis, low immunoglobulin A, bilateral cataracts, anemia, and neurodevelopmental and developmental delay. Patient 3 had severed clinical features with recurrent fever and infections. She was treated with infliximab and symptomatic treatments but without therapeutic effect. She received a stem cell transplantation of umbilical cord blood but died of posttransplant infection and posttransplant graft-vs.-host disease 17 days after transplantation. Finally, a literature review revealed that *TRNT1* variants differed among SIFD patients. Our cases and literature review further expand existing knowledge on the phenotype and *TRNT1* variations of SIFD and suggest that the early genomic diagnosis of *TRNT1* is valuable to promptly assess bone marrow transplantation and tumor necrosis factor inhibitor treatments, which might be effective for the immunodeficiency and inflammation caused by SIFD.

## Introduction

Sideroblastic anemia with B-cell immunodeficiency, periodic fevers, and developmental delay (SIFD) is an autosomal recessive syndrome characterized by severe sideroblastic anemia in the neonatal period or infancy that was first reported in 2013 (1). Next-generation sequencing enables the determination of mutations in the gene nucleotidyltransferase (*TRNT*)-1 (2). *TRNT1* encodes the nucleotidyltransferase tRNA (tRNA-NT) enzyme that catalyzes the addition of the cytosine–cytosine–adenosine (CCA) terminus to the 3′ end of tRNA precursors, which is essential for aminoacylated tRNAs to participate in protein biosynthesis (3, 4). Biallelic *TRNT1* variants impair neuronal cell development and heme synthesis, resulting in SIFD (5), which includes some common clinical features such as sideroblastic anemia, immune deficiency, and periodic fevers. In many individuals with TRNT1 deficiency, the immunoglobulin level is low (hypogammaglobulinemia) (3). To date, only 60 confirmed SIFD cases with *TRNT1* mutations have been described worldwide (3, 5–29). In this study, we report three cases of SIFD resulting from *TRNT1* mutations, including one family with two cases. From these two cases, we identified novel mutations in *TRNT1* (c.1056 + 1G > A and c.1246A > G). In addition, we identified an affected child with severe clinical features. Table 1 shows the clinical features, biochemical detection indices, and genetic analyses of the SIFD patients in this study. Through a systematic review of cases from published articles, we provide new insights for clinicians toward diagnosing SIFD by connecting clinical features and *TRNT1* mutations.

**Table 1:** Clinical features, biochemical detection index, and genetic analysis of SIFD patients (*n* = 3).

Clinical features	Patient 1	Patient 2	Patient 3	
Country of birth	China	China	China	
Gender	Female	Male	Female	
Ethnicity	Han	Han	Han	
Consanguinity	No	No	No	
Age at onset (months)	8	4	3	
Age at diagnosis (months)	116	24	10	
Clinical diagnosis	Recurrent fevers Microcytic anemia Respiratory infection Developmental delay Cerebral atrophy Mild hearing impairment Amyotrophy Microcephaly Cataract Arthritis	Recurrent fevers Microcytic anemia Respiratory infection Diarrhea Developmental delay Amyotrophy Microcephaly Cataract Arthritis	Recurrent fevers Microcytic anemia Respiratory infection Peritonitis Skin soft tissue infection	
Biochemical detection index				Normal reference value
Hb (g/dl)	100 ↓	94 ↓	77↓	110–140
MCV (fl)	67.6 ↓	60.6 ↓	76.7↓	82–100
MCH (pg)	21 ↓	19.1↓	24.6↓	27–34
MCHC (g/L)	311 ↓	315 ↓	321.00	316–354
CRP (mg/L)	33.6 ↑	30.6 ↑	215.0↑	0–8
ESR (mm/h)	24.00	35	NA	0–15
IL-6 (pg/ml0	NA	9.70	21.6↑	0–16.6
TNF-α (pg/ml)	NA	2.10	6.3↑	0–5.2
IgG (g/L)	6.5	8.84	9.73 (after IVIG)	5–10.6
IgA (g/L)	<0.07 ↓	<0.07 ↓	<0.07 ↓	0.13–0.35
IgM (g/L)	0.73	0.56	0.22↓	0.4–1.28
IgE (g/L)	10	12	<5	0–60
C3	0.85	0.97	0.63↓	0.8–1.5
C4	0.19	0.22	0.15	0.12–0.4
**Lymphocyte subsets**				
WBC (10^9^/L)	5.80	8.6	50.5↑	5–12
CD3+	65%	72% ↑	96.99% ↑	39–70%
CD3 + CD4+	43% ↑	53% ↑	67.80% ↑	15–37%
CD3 + CD8+	21%	17%	27.82%	14–39%
CD3-CD19+	19%	17%	0.09% ↓	11–28%
CD3-CD16+/CD56+	12%	9%	2.92% ↓	8–34%
**Therapy**				
Antibiotics	Yes	Yes	Yes	
IVIG	No	Yes	Yes	
TNF-α inhibitor	No	No	Yes	
Stem cell transplantation	No	No	Yes	
Alive/dead	Living	Living	Dead	
Genetic analysis of TRNT1 mutations	c.1056 + 1G > A/c.1246A > G (p.K416E)	c.1056 + 1G > A/c.1246A > G (p.K416E)	c.574C > T (p.Q192*/c.464T > C (p.I155T)	

SIFD, sideroblastic anemia with B-cell immunodeficiency, periodic fevers, and developmental delay; CRP, C-reactive protein; TNF, tumor necrosis factor; IgG, immunoglobulin G; IgA, immunoglobulin A; IgM, immunoglobulin M; IgE, immunoglobulin E; IVIG, intravenous immunoglobulin.

The table shows the statistically significant differences between measurement results and normal range. ↑ The values are higher than normal range. ↓ The values are lower than normal range.

## Case report

**Figure 1:**
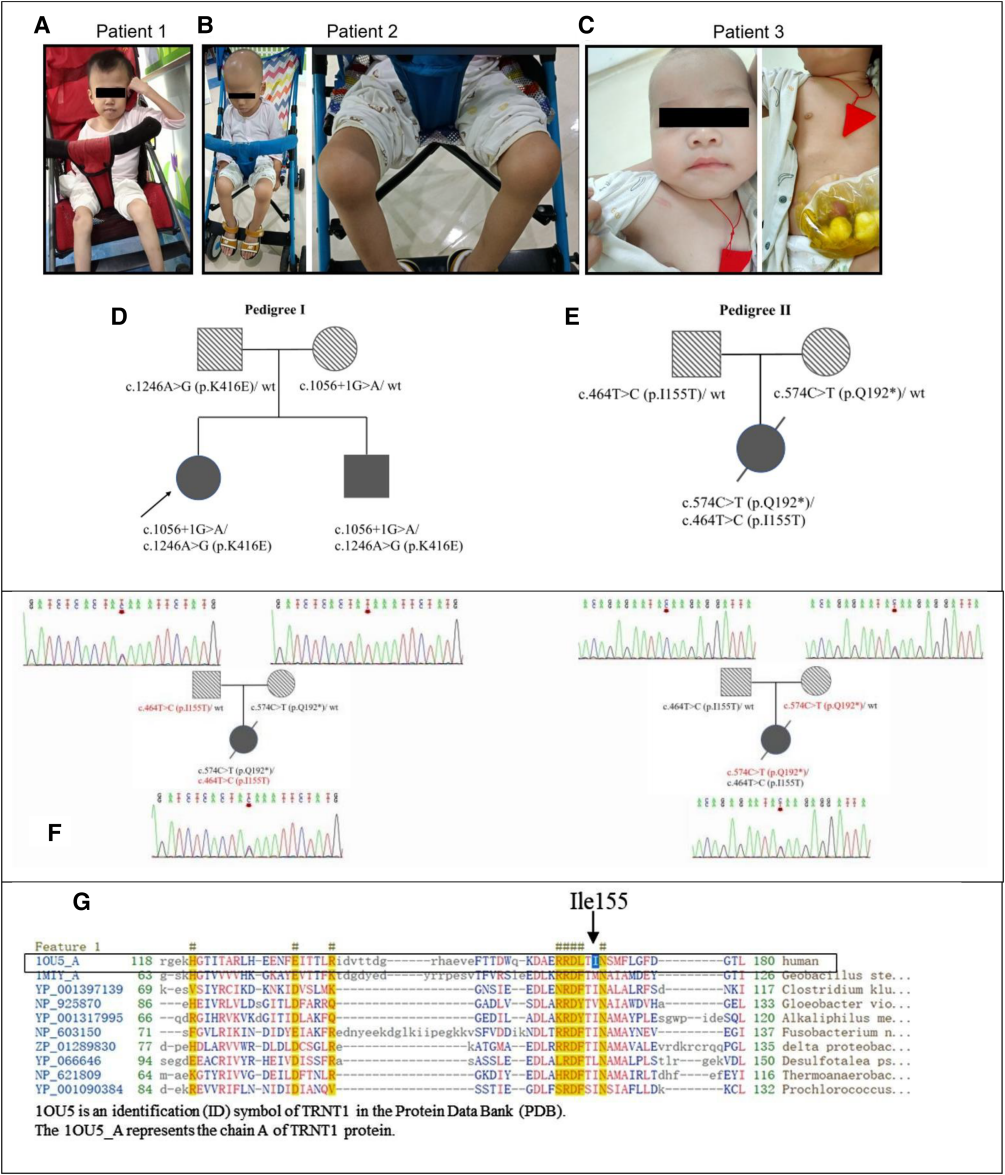
The genetic and clinical characteristics of SIFD patients. (**A**) Patient 1 at the age of 14 years presented with hypotrophic muscle and arthritis. (**B**). Patient 2 at the age of 7 years presented with hypotrophic muscle and arthritis. (**C)** Patient 3 at the age of 11 months. (**D)** The pedigree of siblings in the first family with patient 1 and patient 2. (**E)** The pedigree of patient 3. (**F)** The Sanger sequencing chromatograms of *TRNT1* gene compound heterozygous variations of the family with patient 3. The mutation c.574C > T (p.Q192*) inherited from her mother and c.464T > C (p.I155T) inherited from her father. (**G)** The conserved amino acid sequence in protein TRNT1. The black arrow points a conservative amino acid (Ile155) of protein *TRNT1* in human. SIFD, sideroblastic anemia with B-cell immunodeficiency, periodic fevers, and developmental delay.

Patient 1 (P1), born to non-consanguineous parents, presented with persistent low-grade fever (37.4–38°C) since 8 months of age. The inflammatory markers were elevated every 1–2 months without infective factors, but she recovered following symptomatic treatment. At 16 months of age, she had pain and swelling of her right knee joint with flexion contracture. She could bend her knee and briefly stand but could not walk without hypotonia. At 19 months of age, she was diagnosed with bilateral cataracts. At 7 years of age, cranial magnetic resonance imaging (MRI) showed mild atrophy-like changes in the bilateral cerebral hemispheres and underdeveloped bilateral frontal lobes, suggesting neurodevelopmental delay. The patient had some hearing problems. She could hear louder voice, but no specific hearing test was carried out. She presented with fever several times as an outpatient. She had mild microcytic hypochromic anemia. Sideroblastic anemia could not be determined as her parents did not consent to having her undergo bone marrow aspiration for further examination. Immunoglobulin tests showed a low level of immunoglobulin A (IgA) (<0.07 g/L, range 0.13–0.35 g/L), while immunoglobulin G (IgG), M (IgM), and E (IgE), and complement C3 and C4 were normal. The lymphocyte counting test showed that the ratios and amounts of B- and T-lymphocytes were normal. At this time, she was 16 years old, 12 kg (< −3 SD) in weight, 102 cm (< −3 SD) in height, and had a 48 cm head circumference (Figure 1A). Unfortunately, she did not undergo follow-up visits due to family reasons.

Patient 2 (P2) was the younger male sibling of P1. Similar to his sister, he had unspecified periodic fevers, low levels of IgA, arthritis, and developmental delay; however, he had normal hearing. He was examined routinely at our hospital in the first 3 months after birth. The patient was diagnosed with mild microcytic hypochromic anemia with low levels of IgA (<0.07 g/L, range 0.13–0.35 g/L), whereas neutrophil phagocytosis, B- and T-lymphocyte rates and counts, and IgG were normal. Using ultrasound diagnosis, we found that he had hydrocephalus. At 4 months of age, the patient had diarrhea for the first time for undefined reasons. He was hospitalized at 8 months old with bronchopneumonia and diarrhea. He was diagnosed with a respiratory syncytial virus infection through a pharyngeal swab trial. Thereafter, he experienced febrile episodes every 3–4 weeks, lasting 3–7 days each time. At 19 months of age, he had pain and swelling of the bilateral knee joints and refused to walk on foot. Ultrasonography detection revealed effusions in his knee joints. At 2 years of age, he was diagnosed with bilateral cataracts. Immunological examination showed a slight decrease in IgA, while IgG, IgM, IgE, and B-lymphocyte counts were normal. Afterward, he received intravenous immunoglobulin (IVIG) 400 mg/kg treatment irregularly, resulting in fewer fevers. At 37 months of age, he was hospitalized again due to fever and diarrhea. At 5 years of age, he contracted bronchopneumonia and a type A influenza virus infection. Nevertheless, there were no abnormalities in cardiac and urological ultrasound and cranial MRI detection results. In the same year, his vision was restored after extracapsular cataract extraction with intraocular lens implantation at our hospital. At this point, he was 8 years old, 11 kg (<−3 SD) in weight, 98 cm (<−3 SD) in height, had a 49-cm head circumference (Figure 1B), and could speak simple and short sentences with three to seven words, but the pronunciation was not clear. He stumbled by himself, and fever episodes recurred every 2–3 months.

P1 and her younger brother P2 were treated in our department since May 2016. With the approval of the hospital ethics committee (approval number: 2016021645), peripheral blood was collected from them and their parents for whole-genome sequencing at the Institute of Eugenics and Perinatology at our hospital. The parents had heterozygous mutations in the *TRNT1* gene. Their father had a mutation at c.1246A > G (p.K416E), which has been reported previously (6, 7, 20), whereas their mother had an unreported mutation at c.1056 + 1G > A. The biallelic heterozygous variants of *TRNT1* [c.1056 + 1G > A and c.1246A > G (p.K416E)] were determined in P1 and P2. The c.1056 + 1G > A variant is located at the mRNA splice region in a highly conserved sequence representing functional domains, indicating that the variant may have a structural alteration of the TRNT1 protein, affecting its function. Nevertheless, it has been considered a pathogenic variant with low frequency based on the assessment of the American College of Medical Genetics and Genomics and the Association for Molecular Pathology (ACMG-AMP) (30).

Patient 3 (P3), a Chinese girl, was born from a pair of non-consanguineous Chinese individuals through normal pregnancy (Figure 1C). The mother has a thalassemia trait, but the father is normal. Figures 1D,E show the pedigrees of the two families in this study. Patient 3 presented with a febrile illness and swelling of the skin and soft tissues in the right clavicular region at 3 months of age. The patient was hospitalized to undergo right chest wall debridement and drainage due to bronchopneumonia and necrotic fasciitis. She was hospitalized again at 5 months of age for an abscess in the buttocks and at 6 months of age for bronchopneumonia, perforated sigmoid colon, abdominal adhesions, acute diffuse peritonitis, sepsis, fungal infection, acute suppurative pharyngitis, acute bronchitis, and fat liquefaction in a postoperative wound. Therefore, she underwent a sigmoid colostomy by partially resectioning her colon to repair the perforation. One month later, she was hospitalized again for sepsis, pneumonia, and necrotizing fasciitis in her left big toe. Again, she underwent right chest wall debridement and drainage at 8 months of age when she presented with a fever and cough. Thereafter, she received IVIG treatment every 4 weeks to reduce infection. The compound heterozygous *TRNT1* variant with two functional mutations at c.574C > T (p.Q192*) (inherited from her mother) and c.464T > C (p.I155T) (inherited from her father) was identified through whole-exome sequencing (Figure 1F). No thalassemia gene mutation was found. At 9 months of age, the patient was diagnosed with primary immune deficiency disorders (PID), IgG deficiency, skin and soft tissue infection, iron deficiency anemia, enterocolitis, arthritis, eczema, thrombocytosis, and oral candidiasis. She received IVIG combined with ibuprofen, methylprednisolone, and infliximab to combat inflammation, combined with cephalosporin, vancomycin, meropenem, and fluconazole for anti-infection at another hospital. However, the treatment was ineffective. At 15 months of age, she was hospitalized in the intensive care unit for critical illness. Laboratory tests showed that her white blood cell count was 50.5 × 10^9^/L, neutrophils accounted for 91%, of which band form neutrophilic granulocyte accounted for 47%. No smears were performed at this point; however, the high-sensitivity C-reactive protein (hsCRP) abundance was up to 215 mg/L. *Staphylococcus* infection was detected using a blood culture test. Lymphocyte counts showed B-lymphocyte deficiency and serum immunoglobulin deficiency. A cardiac ultrasound examination showed normal myocardial and intracardiac structure and function. Next, patient 3 was treated with linezolid for bacterial infections. Ring sideroblasts could not be determined without bone marrow aspiration. At 17 months of age, patient 3 underwent umbilical cord blood (UCB) stem cell transplantation. After 5 days of treatment, she had a fever, rash, and mucus discharge from the colostomy. In addition, her inflammatory marker and liver enzyme levels were increased. The results of a blood culture test indicated the patient had a *Stenotrophomonas maltophilia* infection. Taken together, the patient was diagnosed with a posttransplant infection, a posttransplant graft-vs.-host disease, pancytopenia, and metabolic acidosis. Seventeen days after transplantation, the patient died due to severe sepsis and multiorgan failure.

## Discussion

SIFD is a heritable, autosomal recessive disorder with severe multiorgan damage and often results in death during the first decade of life (6). Using whole-exome sequencing, a direct link between *TRNT1* and SIFD was first reported in 2014 (6). *TRNT1* is a nuclear gene encoding the tRNA-NT enzyme, which plays a role in the posttranscriptional modification of tRNAs by adding the CCA trinucleotide to the 3′-end of newly synthesized tRNAs (3, 4). Sasarman et al. suggested that *TRNT1* mutations would impair mitochondrial translation, resulting from defective CCA addition to mitochondrial tRNA^Ser(AGY)^, resulting in an increase in mitochondrial reactive oxygen species that persistently trigger NLRP3 inflammasome activation (12). Recent research suggests that except for previously reported mitochondrial tRNAs, *TRNT1* mutations severely affect the expression of mature cytosolic tRNAs (7). The *TRNT1* mutant cells fail to upregulate protein clearance pathways and perturbations in proteostasis activation in the innate immune system, which results in the overexpression of interleukin-1 (IL-1) and tumor necrosis factor (TNF) (7), suggesting that *TRNT1* mutations are indeed one of the causes of SIFD disease.

TNF inhibitors are typical anti-inflammatory medications used to treat autoimmune diseases such as rheumatoid, juvenile, and psoriatic arthritis, plaque psoriasis, ankylosing spondylitis, ulcerative colitis, and Crohn's disease (31, 32). TNF inhibitors, which could inhibit proinflammatory cytokines in tissues and blood to reduce fever, blood transfusion demand, and chronic anemia, were first reported to treat SIFD patients in 2019 (7). Giannelou et al. described that three patients received etanercept, and one received infliximab treatment and was followed up from 2 to 12 years (7). It was suggested that TNF inhibitor therapy could effectively suppress fevers and restore inflammatory factors to normal in these patients. In the current study, patient 3 also received the infliximab treatment for anti-inflammation. Unfortunately, the treatment was ineffective. Considering the various clinical features in patients with various *TRNT1* variants, more clinical data are needed to assess the therapeutic effect of TNF inhibitors.

Bone marrow transplants are the only reported effective option (4). Three patients have been reported as having received bone marrow transplants. One patient was transplanted with matched bone marrow from his sibling at 5 months of age. However, this patient died following significant neurological complications 38 weeks posttransplant (10). Another patient underwent a myeloablative allogeneic bone marrow transplantation at 9 months of age, remaining healthy over 3 years posttransplantation, except for pigmentary retinitis that occurred 32 months posttransplantation (1). The last patient showed no systemic symptoms 3 years posttransplantation but had moderate hearing loss and retinopathy (4). In the present report, patient 3 underwent UCB stem cell transplantation. However, the patient died due to *S. maltophilia* infection and transplantation-related complications.

To date, 60 SIFD patients with *TRNT1* variants have been reported in previous publications (3, 5–29). In Table 2, we have summarized all the *TRNT1* variants including the three new cases. Through this work, the summarized *TRNT1* mutations can be used by other researchers or clinicians to further investigate their functions in SIFD disease. Patients with the c.1246A > G mutation in *TRNT1* exhibited typical clinical features such as periodic fevers, sideroblastic anemia, developmental delay, diarrhea, bilateral hearing loss, bilateral cataracts, recurrent swelling of digits in hands and knees, and hypotrophic muscle (6, 7, 20). In the present study, the siblings with newly reported *TRNT1* mutations (c.1246A > G and c.1056 + 1G > A) had similar clinical features, including periodic fevers, mild anemia, developmental delay, bilateral cataracts, recurrent swelling of knees, and hypotrophic muscle. Considering the unreported *TRNT1* mutation (c.1056 + 1G > A) is located in the mRNA splice region within a highly conserved sequence, this variant might have more impact and requires more studies in the future.

**Table 2:** Reported pathogenetic variants in *TRNT1*.

Reference	No	Single-nucleotide	Protein variant 1	Type of mutation	Single-nucleotide	Protein variant 2	Type of mutation
		Variant 1			Variant 2		
(3)	1	c.668T > C	p.I223T	Missense	c.342 + 5G > T	NA	Splicing
	2	c.668T > C	p.I223T	Missense	c.342 + 5G > T	NA	Splicing
(5)	3	c.525delT	p.L176*	Nonsense	c.938T > C	p.L313S	Missense
(6)	4	c.569G > T	p.R190I	Missense	c.569G > T	p.R190I	Missense
	5	c.569G > T	p.R190I	Missense	c.569G > T	p.R190I	Missense
	6	c.569G > T	p.R190I	Missense	c.569G > T	p.R190I	Missense
	7	c.668T > C	p.I223T	Missense	c.668T > C	p.I223T	Missense
	8	c.668T > C	p.I223T	Missense	c.1057-7C > G	NA	Splicing
	9	c.668T > C	p.I223T	Missense	c.1057-7C > G	NA	Splicing
	10	c.668T > C	p.I223T	Missense	c.1057-7C > G	NA	Splicing
	11	c.668T > C	p.I223T	Missense	c.218_219ins22	NA	Frameshift
	12	c.668T > C	p.I223T	Missense	c.1142insATGT	p.W381fs	Frameshift
	13	c.668T > C	p.I223T	Missense	c.1252_1253insA	p.S418fs	Frameshift
	14	c.668T > C	p.I223T	Missense	No mutation detected	NA	NA
	15	c.1246A > G	p.K416E	Missense	c. del1054_1056 + 10	NA	Splicing
	16	c.1246A > G	p.K416E	Missense	c. del1054_1056 + 10	NA	Splicing
	17	c.608 + 1 G > T	NA	Splicing	c.461C > T	p.T154I	Missense
	18	c.977T > C	p.I326T	Missense	c.472A > G	p.M158V	Missense
	19	c.497T > C	p.L166S	Missense	c.461C > T	p.T154I	Missense
(7)	20	c.644A > G	p.H215R	Missense	c.644A > G	p.H215R	Missense
	21	c.644A > G	p.H215R	Missense	c.644A > G	p.H215R	Missense
	22	c.295C > T	p.R99W	Missense	c.488A > T	p.D163V	Missense
	23	c.295C > T	p.R99W	Missense	c.488A > T	p.D163V	Missense
	24	c.329C > T	p.T110I	Missense	c.383A > G	p.D128G	Missense
	25	c.329C > T	p.T110I	Missense	c.383A > G	p.D128G	Missense
	26	c.488A > T	p.D163V	Missense	c.668T > C	p.I223T	Missense
	27	c.1246A > G	p.K416E	Missense	c.1245_1246insA	p.S418Kfs*9	Frameshift
	28	c.668T > C	p.I223T	Missense	c.1245_1246insA	p.S418Kfs*9	Frameshift
(8)	29	c.295C > T	p.R99W	Missense	c.295C > T	p.R99W	Missense
	30	c.295C > T	p.R99W	Missense	c.295C > T	p.R99W	Missense
	31	c.295C > T	p.R99W	Missense	c.295C > T	p.R99W	Missense
(9)	32	c.1246A	p.S418fs	Frameshift	c.609-26 T > C	NA	Splicing
	33	c.1246A	p.S418fs	Frameshift	c.609-26 T > C	NA	Splicing
	34	c.1246A	p.S418fs	Frameshift	c.126_128delAGA	p.E43del	Frameshift
(10)	35	c.608 + 1 G > T	NA	Splicing	c.668T > C	p.I223T	Missense
	36	c.608 + 1 G > T	NA	Splicing	c.668T > C	p.I223T	Missense
(11)	37	c.218_219ins22	p.I223T	Frameshift	c.218_219ins22	p.I223T	Frameshift
	38	c.977T > C	p.I326T	Missense	c.977T > C	p.I326T	Missense
(12)	39	c.443C > T	p.A148V	Missense	c.443C > T	p.A148V	Missense
	40	c.383A > G	p.D128G	Missense	c.518A > T	p.Y173F	Missense
(13)	41	c.295C > T	p.R99W	Missense	c.295C > T	p.R99W	Missense
(14)	42	c.295C > T	p.R99W	Missense	c.1234C > T	p.R412*	Nonsense
(15)	43	c.498_501delATTT	p.F167fs	Nonsense	c.947C > T	p.A316V	Missense
(16)	44	NA	NA		NA	NA	
(17)	45	c.565T > C	p.I155T	Missense	c.608G > A	p.R203K	Missense
(18)	46	c.1213G > A	p.G405R	Missense	c.1057-7C > G	NA	Splicing
(19)	47	c.977T > C	p.I326T	Missense	c.977T > C	p.I326T	Missense
	48	c.1213G > A	p.G405R	Missense	c.1057-7C > G	NA	Splicing
(20)	49	c.938delT	p.L313fs	Frameshift	c.1246A > G	p.K416E	Missense
	50	c.608G > A	p.R203K	Missense	c.1246A > G	p.K416E	Missense
(21)	51	c.1057-7C > G	NA	Splicing	c.1092A > T	p.E364D	Missense
	52	c.1057-7C > G	NA	Splicing	c.1092A > T	p.E364D	Missense
(22)	53	c.668T > C	p.I223T	Missense	c.1057-7C > G	NA	Splicing
(23)	54	c.914A > T	p.D305V	Missense	c.914A > T	p.D305V	Missense
(24)	55	c.361 G > A	p.E121K	Missense	c.407 C > G	p.A136G	Missense
(25)	56	c.495_498del	p.F167Tfs*9	Frameshift	c.1246A > G	p.K416E	Missense
(26)	57	c.88A > G	p.M30V	Missense	c.363G > T	p.E121D	Missense
	58	c.302 T > C	p.I101T	Missense	c.1234C > T	p.R412*	Nonsense
(27)	59	c.383A > G	p.D128G	Missense	c.1168G > A	p.G390S	Missense
(28)	60	c.948-949delAAinsGG	p.K317E	Missense	c.948-949delAAinsGG	p.K317E	Missense
This study	61	c.1246A > G	p.K416E	Missense	c.1056 + 1G > A	NA	Splicing
	62	c.1246A > G	p.K416E	Missense	c.1056 + 1G > A	NA	Splicing
	63	c.574C > T	p.Q192*	Nonsense	c.464T > C	p.I155T	Missense

NA, not available.

**Figure 2:**
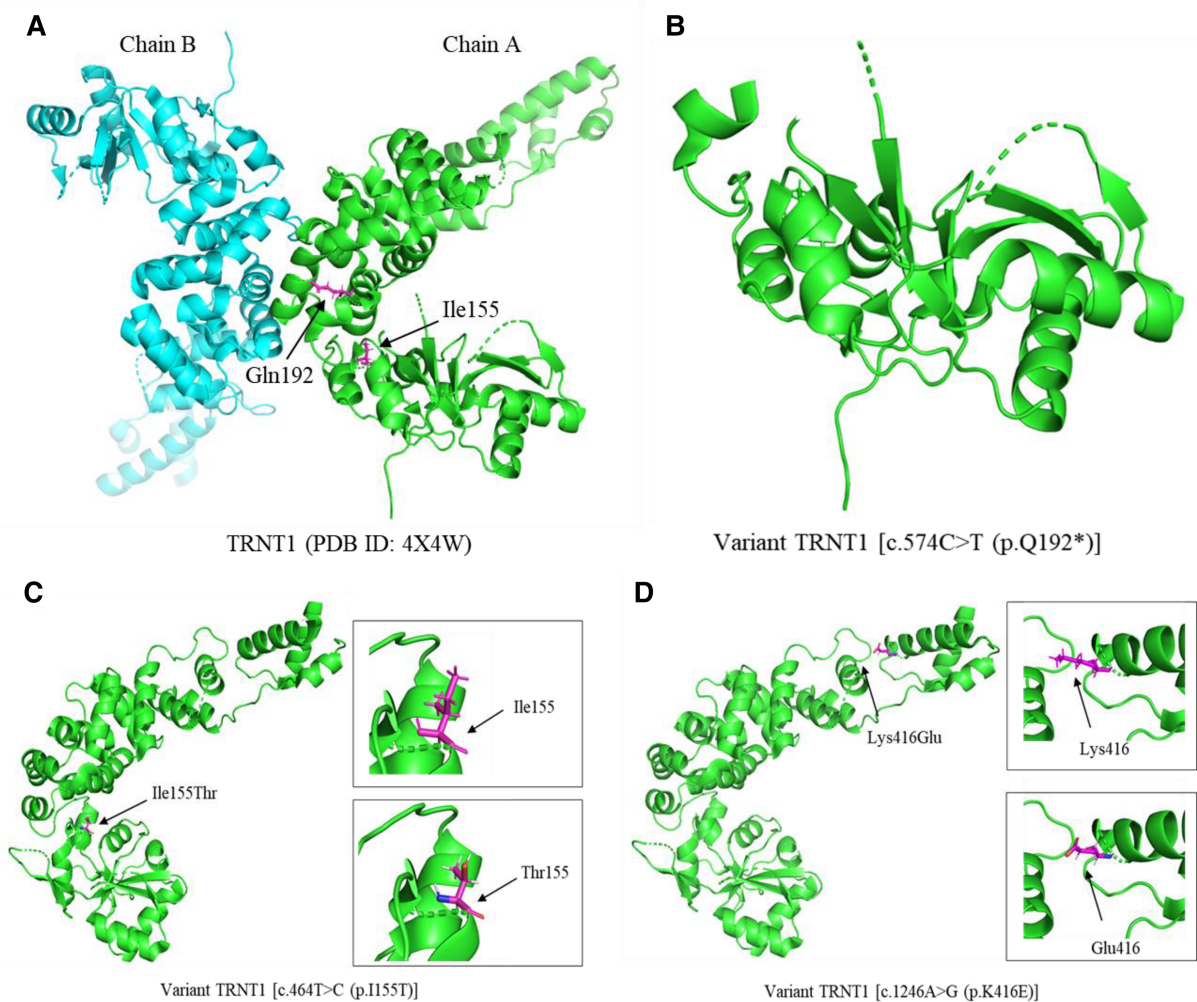
The cartoon diagram of 3D protein structure of TRNT1 and its variants. (**A)** The resolved human protein structure of TRNT1 with its PDB identification (ID). (**B)** The protein structure of variant TRNT1 [c.574C > T (p.Q192*)]. (**C)** The protein structure of variant TRNT1 [c.464T > C (p.I155T)]. The black arrows point the amino acids isoleucine (Ile) and threonine (Thr) at the site of 155. (**D)** The protein structure of variant TRNT1 [c.1246A > G (p.K416E)]. The black arrows point the amino acids lysine (Lys) and glutamate (Glu) at the site of 416. PDB, Protein Data Bank; ID, identification.

Patient 3 with *TRNT1* mutations [c.574C > T (p.Q192*/c.464T > C (p.I155T)] had severe clinical features, including recurrent fevers, immune deficiency disorders, and developmental delay. The resolved human protein structure of TRNT1 with its Protein Data Bank (PDB) identification (ID) is shown in Figure 2A. The c.464T > C (p.I155T) mutation in *TRNT1* affects the conservative amino acid sequence of this protein, which may contribute to protein dysfunction (Figures 1G, 2C). Most importantly, the novel c.574C > T (p.Q192*) mutation causes protein truncation (Figure 2B), which may contribute to the dysfunction of TRNT1. Though we have no evidence to prove the function of truncated TRNT1 in mitochondrial, combined with the biallelic mutations, the dysfunctional TRNT1 protein may have finally led patient 3 to acquire SIFD. Therefore, alteration of TRNT1 protein structure by c.574C > T (p.Q192*) and c.464T > C (p.I155T) mutations may indicate a poor prognosis for patients with SIFD. In the present study, the c.574C > T mutation is reported for the first time. Other studies on this mutation could not be found. Therefore, its function in SIFD deserves to be further explored in the future. Besides the two aforementioned *TRNT1* mutations, the previously reported c.1246A > G (p.K416E) mutation could also produce dysfunctional proteins in patients 1–2 (Figure 2D) (6). Therefore, future studies should consider more effective treatments to correct this mutation, such as gene editing and repair.

The literature review and this study found 63 SIFD patients with *TRNT1* mutations (3, 5–29). However, clinical characteristics were not reported in 16 cases (6). Therefore, we summarized the clinical features of 47 patients in Table 3. These patients have a very varied phenotypic appearance. Developmental delay (66%) and recurrent fevers (68%) are the most common clinical features in SIFD patients with *TRNT1* mutations. The majority of patients have sideroblastic anemia (55%). Cataracts are found in 30% of patients, hearing loss in 21%, diarrhea in 34%, skin and subcutaneous diseases in 26%, and poor balance in 17%. Some SIFD patients also exhibit several features like ataxia, hypotonia, splenomegaly, retinitis pigmentosa, seizures, and cardiomyopathy (3, 5–29).

**Table 3:** Clinical features in patients with *TRNT1* mutations.

Clinical features	% of 47 patients	% of 14 deaths
Developmental delay	66% (31/47)	36% (5/14)
Recurrent fevers	68% (32/47)	71% (10/14)
Sideroblastic anemia	55% (26/47)	86% (12/14)
Cataracts	30% (14/47)	7% (1/14)
Hearing loss	21% (10/47)	14% (2/14)
Diarrhea	34% (16/47)	7% (1/14)
Skin and subcutaneous diseases	26% (12/47)	29% (4/14)
Poor balance	17% (8/47)	0% (0/14)
Total mortality rate	29.8% (14/47)	

## Conclusion

Due to the close association between *TRNT1* variants and SIFD disease, the early detection of *TRNT1* mutations in patients with SIFD would help them get timely treatment. In the present study, we reported three SIFD cases with newly discovered mutations (c.1056 + 1G > A and c.574C > T) in *TRNT1*. Bone marrow transplantation and TNF inhibitor therapy for patients with SIFD may greatly relieve disease symptoms and meet clinical demands.

The inadequacies of this article are that the laboratory testings were not comprehensive for some reasons. The siblings of patients 1 and 2 failed to return to the hospital for further consultation due to family reasons, which led to lose effective treatment measures, and could not evaluate the prognosis appropriately. Also, maybe we need try more measures such as etanercept, colchicine, and anakinra when infliximab treatment failed before stem cell transplantation in patient 3. In addition, we did not carry out the functional study of the new mutations in *TRNT1*, hoping that subsequent studies can clarify the relationship between molecular mechanisms and clinical phenotypes in the future.

## Data Availability

The original contributions presented in the study are included in the article/**Supplementary Material**, further inquiries can be directed to the corresponding author.

## References

[1] Wiseman DH May A Jolles S Connor P Powell C Heeney MM A novel syndrome of congenital sideroblastic anemia, B-cell immunodeficiency, periodic fevers, and developmental delay (SIFD). Blood. (2013) 122(1):112–23. 10.1182/blood-2012-08-439083 23553769 PMC3761334

[2] Yohe S Thyagarajan B. Review of clinical next-generation sequencing. Arch Pathol Lab Med. (2017) 141(11):1544–57. 10.5858/arpa 28782984

[3] Wedatilake Y Niazi R Fassone E Powell CA Pearce S Plagnol V TRNT1 deficiency: clinical, biochemical and molecular genetic features. Orphanet J Rare Dis. (2016) 11(1):90. 10.1186/s13023-016-0477-0 27370603 PMC4930608

[4] Hou YM. CCA Addition to tRNA: implications for tRNA quality control. IUBMB Life. (2010) 62(4):251–60. 10.1002/iub.301 20101632 PMC2848691

[5] Yang L Xue X Zeng T Chen X Zhao Q Tang X Novel biallelic TRNT1 mutations lead to atypical SIFD and multiple immune defects. Genes Dis. (2020) 7(1):128–37. 10.1016/j.gendis.2020.01.005 32181284 PMC7063413

[6] Chakraborty PK Schmitz-Abe K Kennedy EK Mamady H Naas T Durie D Mutations in TRNT1 cause congenital sideroblastic anemia with immunodeficiency, fevers, and developmental delay (SIFD). Blood. (2014) 124(18):2867–71. 10.1182/blood-2014-08-591370 25193871 PMC4215314

[7] Giannelou A Wang H Zhou Q Park YH Abu-Asab MS Ylaya K Aberrant tRNA processing causes an autoinflammatory syndrome responsive to TNF inhibitors. Ann Rheum Dis. (2018) 77(4):612–9. 10.1136/annrheumdis-2017-212401 29358286 PMC5890629

[8] Hull S Malik AN Arno G Mackay DS Plagnol V Michaelides M Expanding the phenotype of TRNT1-related immunodeficiency to include childhood cataract and inner retinal dysfunction. JAMA Ophthalmol. (2016) 134(9):1049–53. 10.1001/jamaophthalmol.2015.5833 27389523

[9] DeLuca AP Whitmore SS Barnes J Sharma TP Westfall TA Scott CA Hypomorphic mutations in TRNT1 cause retinitis pigmentosa with erythrocytic microcytosis. Hum Mol Genet. (2016) 25(1):44–56. 10.1093/hmg/ddv446 26494905 PMC4690490

[10] Barton C Kausar S Kerr D Bitetti S Wynn R. SIFD As a novel cause of severe fetal hydrops and neonatal anaemia with iron loading and marked extramedullary haemopoiesis. J Clin Pathol. (2018) 71(3):275–8. 10.1136/jclinpath-2017-204698 29055896 PMC5868532

[11] Fouquet C Le Rouzic MA Leblanc T Fouyssac F Leverger G Hessissen L Genotype/phenotype correlations of childhood-onset congenital sideroblastic anaemia in a European cohort. Br J Haematol. (2019) 187(4):530–42. 10.1111/bjh.16100 31338833

[12] Sasarman F Thiffault I Weraarpachai W Salomon S Maftei C Gauthier J The 3’ addition of CCA to mitochondrial tRNASer(AGY) is specifically impaired in patients with mutations in the tRNA nucleotidyl transferase TRNT1. Hum Mol Genet. (2015) 24(10):2841–7. 10.1093/hmg/ddv044 25652405 PMC4406295

[13] Frans G Moens L Schaballie H Wuyts G Liston A Poesen K Homozygous N-terminal missense mutation in TRNT1 leads to progressive B-cell immunodeficiency in adulthood. J Allergy Clin Immunol. (2017) 139(1):360–363.e6. 10.1016/j.jaci.2016.06.050 27531075

[14] Kumaki E Tanaka K Imai K Aoki-Nogami Y Ishiguro A Okada S Atypical SIFD with novel TRNT1 mutations: a case study on the pathogenesis of B-cell deficiency. Int J Hematol. (2019) 109(4):382–9. 10.1007/s12185-019-02614-0 30758723

[15] Rigante D Stellacci E Leoni C Onesimo R Radio FC Pizzi S Biallelic TRNT1 variants in a child with B cell immunodeficiency, periodic fever and developmental delay without sideroblastic anemia (SIFD variant). Immunol Lett. (2020) 225:64–5. 10.1016/j.imlet.2020.06.012 32592741

[16] Hoang TK Albert DA. Novel presentations of periodic fever syndromes: discrepancies between genetic and clinical diagnoses. Eur J Rheumatol. (2019) 6(1):12–8. 10.5152/eurjrheum.2018.18023 30407166 PMC6459325

[17] Lougaris V Chou J Baronio M Gazzurelli L Lorenzini T Soresina A Novel biallelic TRNT1 mutations resulting in sideroblastic anemia, combined B and T cell defects, hypogammaglobulinemia, recurrent infections, hypertrophic cardiomyopathy and developmental delay. Clin Immunol. (2018) 188:20–2. 10.1016/j.clim.2017.11.008 29170023

[18] Bader-Meunier B Rieux-Laucat F Touzot F Frémond ML André-Schmutz I Fraitag S Inherited immunodeficiency: a new association with early-onset childhood panniculitis. Pediatrics. (2018) 141(Suppl 5):S496–500. 10.1542/peds.2017-0213 29610179

[19] Frémond ML Melki I Kracker S Bondet V Duffy D Rice GI Comment on: ‘aberrant tRNA processing causes an autoinflammatory syndrome responsive to TNF inhibitors’ by Giannelou et al: mutations in TRNT1 result in a constitutive activation of type I interferon signalling. Ann Rheum Dis. (2019) 78(8):e86. 10.1136/annrheumdis-2018-213745 29858171

[20] Orlando F Naddei R Stellacci E Gallinoro CM Melis D Tartaglia M Etanercept as a successful therapy in autoinflammatory syndrome related to TRNT1 mutations: a case-based review. Clin Rheumatol. (2021) 40(10):4341–8. 10.1007/s10067-021-05653-3 33646446

[21] Odom J Amin H Gijavanekar C Elsea SH Kralik S Chinen J A phenotypic expansion of TRNT1 associated sideroblastic anemia with immunodeficiency, fevers, and developmental delay. Am J Med Genet A. (2022) 188(1):259–68. 10.1002/ajmg.a.62482 34510712

[22] Jfri A El-Helou T Watters KA Bélisle A Litvinov IV Netchiporouk E. Congenital sideroblastic anemia associated with B cell immunodeficiency, periodic fevers, and developmental delay: a case report and review of mucocutaneous features. SAGE Open Med Case Rep. (2019) 7:2050313X19876710. 10.1177/2050313X19876710 31555444 PMC6747858

[23] Topyildiz E Edeer Karaca N Bas I Aykut A Durmaz A Guven Bilgin RB A novel homozygous TRNT1 mutation in a child with an early diagnosis of common variable immunodeficiency leading to mild hypogammaglobulinemia and hemolytic anemia. J Pediatr Hematol Oncol. (2021) 43(6):e780–4. 10.1097/MPH.0000000000002101 33843817

[24] Mendonca LO Prado AI Costa IMC Bandeira M Dyer R Barros SF Case report: expanding clinical, immunological and genetic findings in sideroblastic anemia with immunodeficiency, fevers and development delay (SIFD) syndrome. Front Immunol. (2021) 12:586320. 10.3389/fimmu.2021.586320 33936027 PMC8079983

[25] Bardou MLD Rivitti-Machado MC Michalany NS de Jesus AA Goldbach-Mansky R Barros JCR Neutrophilic dermatosis: a new skin manifestation and novel pathogenic variant in a rare autoinflammatory disease. Australas J Dermatol. (2021) 62(2):e276–9. 10.1111/ajd.13527 33332575

[26] Wang J Deng Q He X Chen D Hang S Gao Y Two cases of sideroblastic anemia with B-cell immunodeficiency, periodic fevers, and developmental delay (SIFD) syndrome in Chinese Han children caused by novel compound heterozygous variants of the TRNT1 gene. Clin Chim Acta. (2021) 521:244–50. 10.1016/j.cca.2021.07.019 34310935

[27] Maccora I Ramanan AV Vergnano S Roderick MR. Sideroblastic anaemia, immunodeficiency, periodic fevers and developmental delay (SIFD) presenting as systemic inflammation with arthritis. Rheumatology (Oxford). (2021) 60(7):e234–6. 10.1093/rheumatology/keab010.31 33493307

[28] Kisla Ekinci RM Zararsiz A Demir GU Anlas O. A rare autoinflammatory disorder in a pediatric patient with favorable response to etanercept: sideroblastic anemia with B cell immunodeficiency, periodic fevers, and developmental delay syndrome. Pediatr Allergy Immunol Pulmonol. (2022) 35(3):129–32. 10.1089/ped.2022.0090 36121781

[29] Maccora I Ramanan AV Wiseman D Marrani E Mastrolia MV Simonini G. Clinical and therapeutic aspects of sideroblastic anaemia with B-cell immunodeficiency, periodic fever and developmental delay (SIFD) syndrome: a systematic review. J Clin Immunol. (2023) 43(1):1–30. 10.1007/s10875-022-01343-0 35984545 PMC9840570

[30] Richards S Aziz N Bale S Bick D Das S Gastier-Foster J Standards and guidelines for the interpretation of sequence variants: a joint consensus recommendation of the American College of Medical Genetics and Genomics and the Association for Molecular Pathology. Genet Med. (2015) 17(5):405–24. 10.1038/gim.2015.30 25741868 PMC4544753

[31] Willrich MA Murray DL Snyder MR. Tumor necrosis factor inhibitors: clinical utility in autoimmune diseases. Transl Res. (2015) 165(2):270–82. 10.1016/j.trsl.2014.09.006 25305470

[32] Jang DI Lee AH Shin HY Song HR Park JH Kang TB The role of tumor necrosis factor alpha (TNF-α) in autoimmune disease and current TNF-α inhibitors in therapeutics. Int J Mol Sci. (2021) 22(5):2719. 10.3390/ijms22052719 33800290 PMC7962638

