# Supplementary material for: Case report: Sideroblastic anemia with B-cell immunodeficiency, periodic fevers, and developmental delay: Three cases and a literature review
Source: Front Pediatr. 2023 Mar 2;11:1001222. doi: 10.3389/fped.2023.1001222 (PMC10017860; doi:10.3389/fped.2023.1001222)
Supplement: Supplementary file 1 [file Table1.docx]

SUPPLEMENT materials

Case 1 (P1) was the first baby who was born to the family without history of consanguinity in 2006. She was delivered by the mother at 39 weeks of gestation. Her birth weight was 2.8Kg, and the hearing screening of both ears was normal.The little girl presented persistent low-grade fevers (37.4−38°C) since the age of 8 months, occurring every 1-2 months with elevated inflammatory markers. No infective cause was found and she recovered following symptomatic treatment. At 16-month-old, she had pain and swelling of her right knee joint, and presented with flexion contracture. She had no hypotonia and was unable to walk, but she was able to bend her knee and briefly stand. At 19-month-old, she was diagnosed with bilateral cataracts. At 7-year-old, the cranial MRI suggested mild atrophy-like changes in the bilateral cerebral hemispheres, and the bilateral frontal lobes were not fully developed, suggesting a neurodevelopment delay. She visited our outpatient clinic for fever several times. Blood tests suggested mild microcytic hypochromic anemia and peripheral blood iron metabolism was normal. The family did not agree to perform the bone marrow aspiration for further examination, which can’t determine if the girl had sideroblastic anemia. Immunoglobulin tests suggested low immunoglobulin A (IgA) (<0.07 g/l, range 0.13-0.35 g/L), while immunoglobulin G (IgG), immunoglobulin M (IgM), immunoglobulin E (IgE), complement C3 and complement C4 were normal. Lymphocyte counts showed that B- and T-lymphocyte ratios and counts were normal. She is now 16-year-old, 12 kg (< -3SD) in weight, 102 cm (< -3SD) tall, and has a head circumference of 48 cm (Fig. 1 a). She didn’t attend the follow-up visits due to the family reasons.

Case 2 (P2), was the younger male sibling of P1, born to the same parents in 2014. He was delivered by the mother at 38^+5^ weeks of gestation. His birth weight was 3.0Kg. Considering his sister had unknown periodic fevers, immunodeficiency, and developmental delay, the boy was examined routinely at our hospital in the 3 months after birth, and the hearing was normal. The boy was diagnosed with mild microcytic hypochromic anemia (Hb 8.7g/dL at 4 months) with low level of IgA (<0.07 g/l, range 0.13-0.35 g/L), while neutrophil phagocytosis, B- and T-lymphocyte rate and counts, and IgG were normal. The ultrasound diagnosed a hydrocephalus. At 4-month-old, the boy had diarrhea for the first time. However, the causes of Diarrhea didn’t be identified. He was hospitalized 8 months after birth with bronchopneumonia, and had diarrhea again. The pharyngeal swab suggested the respiratory syncytial virus infection. After that, he experienced febrile episodes every 3-4 weeks lasting 3-7 days. At 19-month-old, he had pain and swelling of bilateral knee joints, and refused to walk on the ground. Ultrasonography detected joint effusions in his knees. At 2-year-old, he was diagnosed with bilateral cataracts. After that, he was aperiodically given intravenous immunoglobulin (IVIG) therapy and had fewer fevers than before. At 37-month-old, he was hospitalized again for fever and diarrhea, and at 5-year-old for bronchopneumonia and influenza virus type A infection. Cardiac ultrasound, urological ultrasound, and cranial MRI didn’t detect abnormalities. At 5-year-old, his vision was restored after extracapsular cataract extraction with intraocular lens (IOL) implantation in our hospital. He is now 8-year-old, 11Kg(<-3SD) in weight, 98cm(<-3SD) in height, and has a 49cm head circumference (Fig. 1B). He can speak simple short sentences of 3 to 7 words, but the pronunciation is not clear. He was stumble while walking by himself. The episodes of fever recurred every 2 to 3 months now.

Case 3 (P3) was born to unrelated Chinese parents after a normal pregnancy in 2019. Her birth weight was 3.4Kg and was delivered by the mother at 40 weeks of gestation.The mother has thalassemia trait but the father is normal. The girl presented at 3 months of age with a febrile illness and right clavicle swelling. She was hospitalized with bronchopneumonia and necrotic fasciitis, and was performed right chest wall debridement and drainage. She was hospitalized again at 5-month-old for abscess in the buttocks.At 6-month-old,she almost failed to live for severe infection,and was diagnosed with bronchopneumonia, perforated sigmoid colon, abdominal adhesions, acute diffuse peritonitis, sepsis, fungal infection, acute suppurative pharyngitis, acute bronchitis, fat liquefaction in postoperative wound and was performed with partial resection of the sigmoid colon to repair the perforation and created a sigmoid colostomy (Fig. 1C). One month later, she was hospitalized again for sepsis and pneumonia with necrotizing fasciitis in left big toe. She was performed right chest wall debridement and drainage again at 8 months of age. After that, she was received IVIG treatment every 4 weeks and the number of newly infections was decreasing. The whole genome sequencing identified compound heterozygosity for two functionally relevant variants in TRTN1, c.574C>T (p.Gln 192Ter, which was inherited from mother) and c.947C>T (p.Ile 155Thr, which was inherited from father), as the molecular cause underlying the disorder in this child. At 9-month-old, the girl was diagnosed with primary immune deficiency disorders (PID), IgG deficiency (1.05 g/L, normal range 3.5-5.0 g/L), subcutaneous swelling, iron deficiency anemia, enterocolitis, arthritis, eczema, thrombocytosis, and oral candidiasis, and was given IVIG, combination of ibuprofen, methylprednisolone, and infliximab for anti-inflammatory, combination of cephalosperin, vancomycin, meropenem, and fluconazole for anti-infection in other hospital. However, the treatment was ineffective. At 15-month-old, she was hospitalized in ICU for critical illness. Laboratory test showed WBC number was 50.5×10^9^/L, 47% of neutrophils were stab nuclear, High-sensitivity C-reactive protein (hsCRP) was 215mg/L. Blood culture detected staphylococcus. Lymphocyte counts showed presence of lymphocyte subsets: CD19+ (0.09 %, normal range 11-28%), CD3+/CD45+ (96.99%, normal range 39-70%), CD3+CD4+ (67.80%, normal range: 15-37%), CD3+CD8+ (27.82 %, normal range 14-39%), CD16+CD56+ (2.92 %, normal range 8-34%). Immunoglobulin tests suggested low levels of IgA (<0.07 g/L, normal range 0.13-0.35 g/L), IgM (0.22 g/L, normal range 0.63-2.77 g/L) and complement C3 (0.63 g/L, normal range 0.88-2.01 g/L), while levels of IgG (9.73 g/L, normal range 7.23-16.85 g/L) and complement C4 (0.15 g/L, normal range 0.15-0.45 g/L) were normal, complement C3 and are normal. Patient was given Linezolid for bacterial infections. The presence of ring sideroblasts was unable to be determined since bone marrow aspiration wasn’t performed. At 17-month-old, she underwent the umbilical cord blood stem cell transplantation. After 5 days, she presented with fever and a rash. The mucus discharge from the colostomy, inflammatory markers, and liver enzymes were increased. Laboratory test using blood culture indicated the girl had stenotrophomonas maltophilia. Taken together, the girl was diagnosed with post-transplant infection, post-transplant graft-versus-host disease, pancytopenia, metabolic acidosis. 17 days after transplantation, this child passed away due to severe sepsis and multiorgan failure. The patient had developmental delay, normal hearing, no feeding issues, no seizures, and no abnormal lesions on the retina.
